# Algorithms for ribosome traffic engineering and their potential in improving host cells' titer and growth rate

**DOI:** 10.1038/s41598-020-78260-y

**Published:** 2020-12-03

**Authors:** Hadas Zur, Rachel Cohen-Kupiec, Sophie Vinokour, Tamir Tuller

**Affiliations:** 1 Department of Biomedical Engineering, the Engineering Faculty, Tel Aviv University, Tel-Aviv, Israel; 2 The Sagol School of Neuroscience, Tel Aviv University, 69978 Tel-Aviv, Israel

**Keywords:** Biotechnology, Computational biology and bioinformatics, Evolution, Microbiology, Systems biology, Engineering

## Abstract

mRNA translation is a fundamental cellular process consuming most of the intracellular energy; thus, it is under extensive evolutionary selection for optimization, and its efficiency can affect the host's growth rate. We describe a generic approach for improving the growth rate (fitness) of any organism by introducing synonymous mutations based on comprehensive computational models. The algorithms introduce silent mutations that may improve the allocation of ribosomes in the cells via the decreasing of their traffic jams during translation respectively. As a result, resources availability in the cell changes leading to improved growth-rate. We demonstrate experimentally the implementation of the method on *Saccharomyces cerevisiae*: we show that by introducing a few mutations in two computationally selected genes the mutant's titer increased. Our approach can be employed for improving the growth rate of any organism providing the existence of data for inferring models, and with the relevant genomic engineering tools; thus, it is expected to be extremely useful in biotechnology, medicine, and agriculture.

## Introduction

mRNA translation is a central intracellular process consuming most of the cell energy (up to 75%)^1–10^, and is fundamentally relevant to various biomedical phenomena such as genome evolution, human diseases, and intracellular biophysics (see for example^11–24^). Thus, naturally, this process undergoes extensive evolutionary selection for its optimization^25,26^. Moreover, translation optimization is often an important biotechnological objective^2,5–10,26–36^; specifically, when cells are used as a factory for generating proteins (heterologous gene expression) or metabolites, the optimization of the translation process directly affects the efficiency of this factory, yielding a critical economic impact.

It was shown that in both prokaryotes (bacteria and archaea) and eukaryotes, the first ~ 30–50 codons of the ORF tend to be recognized by tRNA species with lower intracellular abundance, resulting in slower ribosomal elongation speed in this region^37,38^. This provides several physiological benefits, such as assisting in ribosomal allocation, recruiting protein chaperons, co-translational folding, and protein maturation^37,38^.

For simplicity, we propose to exploit this region’s properties via introducing silent engineered mutations to the first 50 codons of the endogenous genes, and thereby modulate the free ribosomal pool, while constraining the limits of translation efficiency (reduction or enhancement) of these genes. This is facilitated by the inherent redundancy of the genetic code, where 61 codons encode only 20 amino acids^39,40^, such that we are able to change the elongation rate in this region while maintaining the encoded protein.

The novel approach suggested in this study can be used for biotechnological objectives such as heterologous gene expression and vaccine development.

## Results

### Fitting a whole cell simulation model to experimental data

Our mRNA translation model is based on a mean field approximation of the TASEP (Totally Asymmetric Simple Exclusion Process) which considers all the fundamental aspects of translation dynamics such as: ribosome movement according to codon decoding times from the 5′ to the 3′ end of the mRNA, and excluded volume interactions^41^. In addition, the model includes all the ribosomes and mRNAs in the cell, considers the effect of a finite number of ribosomes in the cell, and the resultant 'competition' between mRNAs for ribosomes^28^. All the model parameters are fitted based on experimental measurements (see "Methods"). We inferred the model for *S. cerevisiae,* and show that the correlation over all mRNAs between ribosomal densities in the model and respective Ribo-seq measurements is 0.85 (p < 10^−308^) (see "Methods").

### Modulating organism fitness based on whole cell simulation

Computational simulation of the whole cell translation model with an order of magnitude of 2 × 10^5^ ribosomes and 6 × 10^4^ mRNA molecules is a challenging time consuming endeavour. Thus, we developed various heuristics for Ribosome Traffic Engineering (RTE); these heuristics are used for efficiently detecting potential mutations that improve/decrease ribosome allocation via the 'deletion'/'introduction' of ribosome traffic jams. As mentioned, we focused on the first 50 codons of the endogenous genes, where evolution tends to introduce relatively slower codons^38^, omitting the first 10 codons due to important regulatory signals in that region^37^. Specifically, the algorithm aimed at mutating codons to their slower or faster synonymous codons, resulting in an increase/decrease in the free ribosomal pool. This in turn, will affect the host growth-rate (fitness).

**Figure 1:**
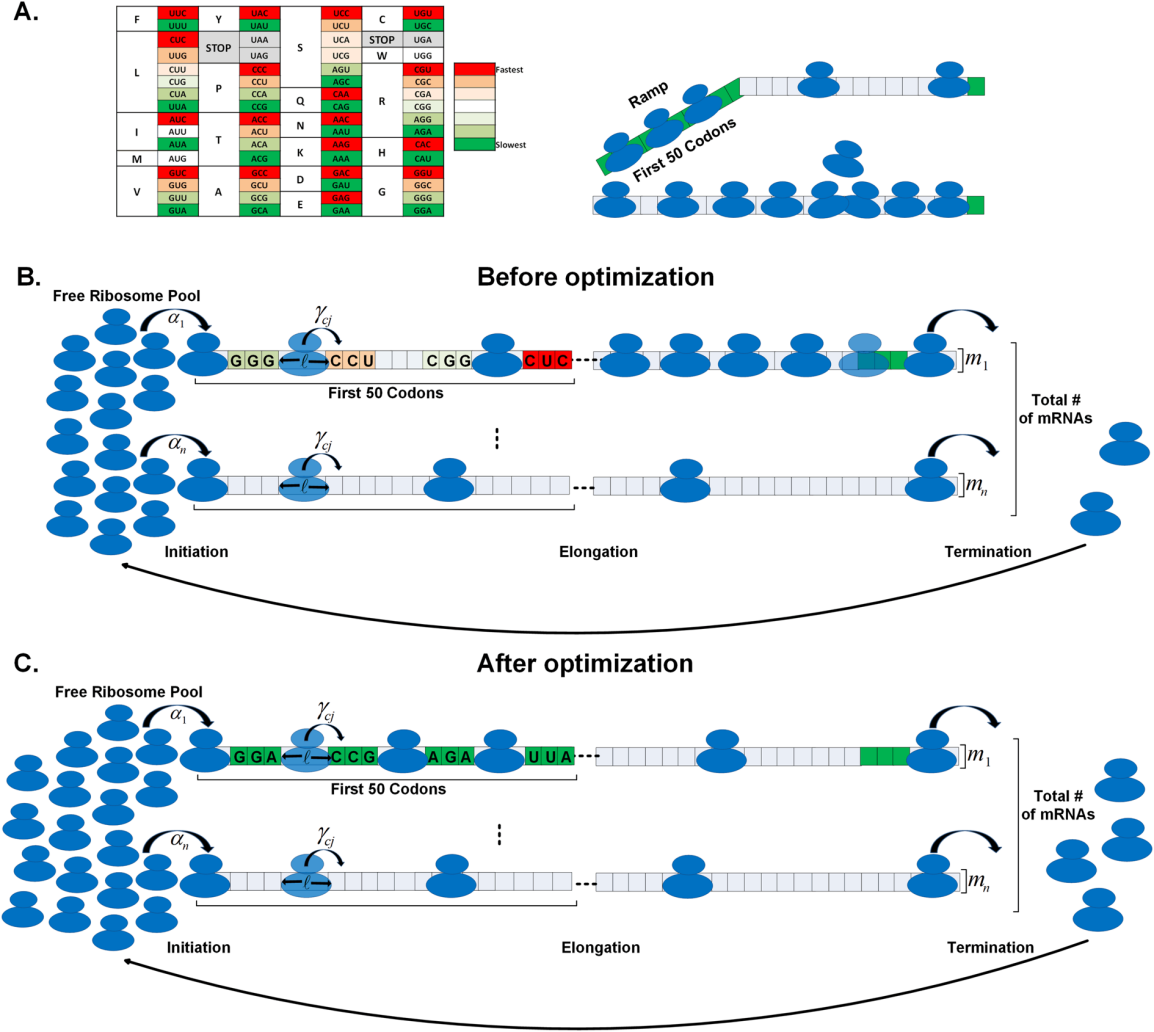
(**A**) Left: The genetic code with per synonymous codons relative speeds (see "Methods" for calculation details) based on the real *S. cerevisiae* genome, the darkest red signifies the fastest relative codon while the darkest green the slowest. Right: An illustration of the ramp (first 50 codons) depicting the benefit of assisting in ribosomal allocation. (**B**) An illustration of the translation simulation before optimization (where in the first iteration of our approach the first gene will be selected to be optimized, see (**C**)), with $\ell$ denoting the ribosome length, $m_{i}$ per gene mRNA levels, $\alpha_{i}$ transcript specific initiation rates, $\gamma_{cj}$ codon specific elongation rates. (**C**) The translation simulation after the first iteration where the first gene was optimized, as illustrated all the codons viable for modification were converted to their slowest synonymous codon. As can be seen as a result of the modifications the number of ribosomes on the first gene is reduced and the free ribosome pool increases.

Briefly (See Fig. 1 and full details in the Methods section), we determine the optimal mutations across the host genome, according to the following RTE greedy algorithm:

Iterate all the host genes, for each gene we look at codons 11–50—a region that we call 'ramp', and mutate a codon to a slower or faster synonymous codon (according to the objective: increase or decrease growth rate). This is done as long as it does not reduce/increase the gene’s translation rate (translation efficiency (TE), see "Methods") beyond some threshold τ; we checked, for example, values between 0.1% and 5% for these thresholds. The best mutation is the one that most increases or decreases the free ribosome pool, and it is selected.

Iterating one mutation at a time across the entire genome is overly time consuming, and also counterproductive, as ultimately we would like to minimize the number of genes we are mutating, to improve the efficiency of the experimental genome engineering steps. Thus, we developed 3 variants of the above approach which operate at the gene level:

1. Forward Gene Minimization (FGM): Per gene start at the beginning of the ORF and incorporate all mutations that improve the free ribosomal pool while not reducing/increasing the gene’s translation rate beyond some threshold τ. In each iteration, the gene that most increases/decreases the free ribosomal pool is selected.
2. Backward Gene Minimization (BGM): Similarly to FGM, only now we start at the end of the modified region in the gene’s ORF and traverse backwards. The logic for this variation is that since many important regulatory signals (some related to initiation regulation) are encoded at the beginning of the ORF^37^ we may want to maintain them.
3. Greedy Gene Minimization (GGM): Per gene iterate over all possible mutations and choose the one that most increases/decreases the free ribosomal pool. Repeat this procedure until no more mutations can be selected without violating the translation rate threshold τ (in the respective direction). Select the gene that most increases/decreases the free ribosomal pool.

One could continue until there is no improvement. Here, for practical reasons, we report the estimated effect after mutating up to 100 genes.

In this scheme a gene can only be mutated to all its free ribosomal pool improving slowest codons (in the improved growth-rate version, the same of-course can be implemented for the reduced). However, could the next best slowest codon also improve the fitness? We tested this scheme, allowing the next best slowest codon to be selected if the slowest one could not be selected due to TE constraint breach, and it did further increase the free ribosomal pool (see following section and Methods).

### Ribosome traffic engineering in 100 genes is estimated to enable improvement of up to 57%/35% in ribosome allocation in *E. coli* and *S. cerevisiae*, respectively

**Figure 2:**
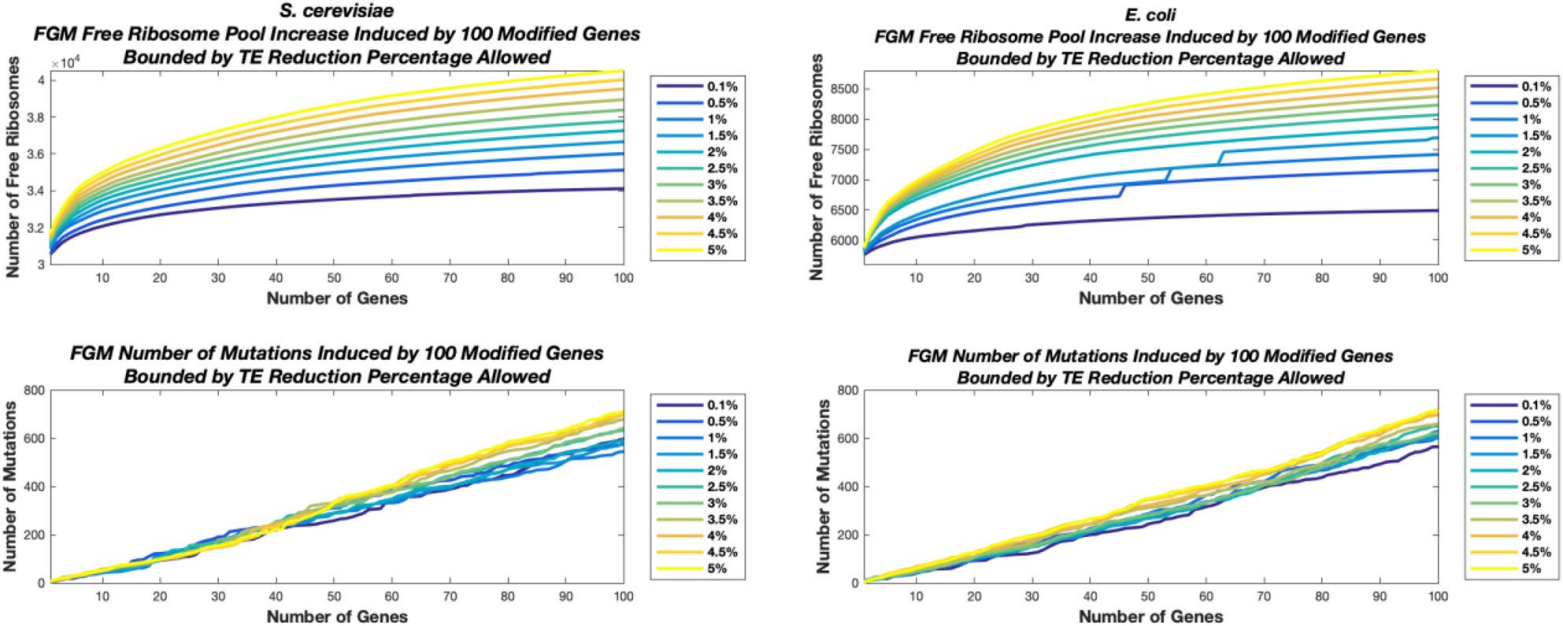
Top two graphs, the baseline free ribosomal pool of *S. cerevisiae* is 30,000 ribosomes. We performed the FGM algorithm for 100 genes, for 11 TE constraints (see legends at the bottom of the figures). As can be seen, the free ribosomal pool steadily increases with each newly modified gene. For Example, for 0.1% reduction in TE the free ribosomal pool after modifying 100 genes is 34,111 with a total of 598 mutations while for 0.5% 35,118 free ribosomes and 575 mutations for 1% 36,012 free ribosomes and 545 mutations, for 1.5% 36,662 free ribosomes and 581 mutations, for 2% 37,261 free ribosomes and 593 mutations, for 2.5% 37,783 free ribosomes and 633 mutations, for 3% 38,380 free ribosomes and 642 mutations, for 3.5% 38,946 free ribosomes and 678 mutations, for 4% 39,529 free ribosomes and 696 mutations, for 4.5% 40,024 free ribosomes and 699 mutations, for 5% 40,517 free ribosomes and 710 mutations. Bottom two graphs, the baseline free ribosomal pool of *E. coli* is 5600 ribosomes. We performed the FGM algorithm for 100 genes, for 11 TE constraints. As can be seen the free ribosomal pool steadily increases with each newly modified gene. For example, for 0.1% reduction in TE the free ribosomal pool after modifying 100 genes is 6,490 with 565 mutations while for 0.5% 7,154 free ribosomes and 605 mutations for 1% 7,415 free ribosomes and 629 mutations, for 1.5% 7691 free ribosomes and 601 mutations, for 2% 7861 free ribosomes and 616 mutations, for 2.5% 8,071 free ribosomes and 650 mutations, for 3% 8,231 free ribosomes and 622 mutations, for 3.5% 8375 free ribosomes and 660 mutations, for 4% 8,516 free ribosomes and 697 mutations, for 4.5% 8,661 free ribosomes and 715 mutations, for 5% 8,799 free ribosomes and 720 mutations. See also Table 1 and supplementary table S1 for a summary of the results.

As can be seen in Fig. 2 for *S. cerevisiae* and *E. coli*, utilizing the FGM variant of our RTE approach (results for BGM and GGM can be found in the Supplementary), the free ribosomal pool steadily increases with each newly modified gene.

Table 1 summarizes the number of additional free ribosomes each of the 3 algorithms enables according to the TE reduction constraint in *S. cerevisiae*, and in *E. coli*, along with the free ribosomal pool percentage increase, and mean number of mutations performed across the top 10 selected genes, allowing the next best synonymous mutation to be selected. See also supplementary table S1.

**Table 1:** The table summarizes the additional number of free ribosomes made available by each of the three algorithms for *S. cerevisiae (A.)* and for *E. coli (B.)*.

Reduction in TE (%)	FGM Free Ribosomes	BGM Free Ribosomes	GGM Free Ribosomes
**A**			
0.1	2076 (6.92%) [4.10]	1248 (4.16%) [3.10]	1217 (4.06%) [1.10]
0.5	2418 (8.06%) [4.20]	2103 (7.01%) [3.60]	1774 (5.91%) [1.50]
1	2895 (9.65%) [4.20]	2390 (7.97%) [4.30]	1929 (6.43%) [1.40]
1.5	3209 (10.70%) [4.30]	2624 (8.75%) [4.50]	2778 (9.26%) [1.80]
2	3496 (11.65%) [5.00]	2850 (9.50%) [4.00]	3090 (10.30%) [1.60]
2.5	3741 (12.47%) [5.70]	3056 (10.19%) [4.50]	3360 (11.20%) [2.10]
3	3966 (13.22%) [5.60]	3264 (10.88%) [5.10]	3677 (12.26%) [2.40]
3.5	4196 (13.99%) [5.80]	3460 (11.53%) [5.10]	3818 (12.73%) [2.00]
4	4468 (14.89%) [5.70]	3630 (12.10%) [4.60]	4062 (13.54%) [2.10]
4.5	4743 (15.81%) [4.90]	3894 (12.98%) [3.90]	4367 (14.56%) [2.30]
5	4994 (16.65%) [5.20]	4066 (13.55%) [4.70]	4523 (15.08%) [2.20]
**B**			
0.1	449 (8.01%) [4.00]	414 (7.39%) [4.40]	471 (8.42%) [2.40]
0.5	642 (11.47%) [4.40]	756 (13.49%) [5.10]	805 (14.38%) [2.70]
1	740 (13.22%) [4.40]	843 (15.06%) [5.30]	918 (16.40%) [2.50]
1.5	812 (14.50%) [4.80]	928 (16.57%) [5.80]	986 (17.61%) [2.60]
2	1073 (19.17%) [4.40]	984 (17.58%) [6.40]	1059 (18.91%) [2.70]
2.5	1139 (20.33%) [4.40]	1025 (18.31%) [7.20]	1113 (19.87%) [2.80]
3	1191 (21.27%) [4.80]	1078 (19.25%) [6.60]	1177 (21.02%) [3.10]
3.5	1236 (22.07%) [5.30]	1126 (20.11%) [7.60]	1233 (22.01%) [3.00]
4	1284 (22.92%) [6.30]	1157 (20.66%) [6.70]	1258 (22.46%) [2.90]
4.5	1325 (23.66%) [6.90]	1188 (21.21%) [6.80]	1303 (23.27%) [2.90]
5	1371 (24.49%) [6.20]	1231 (21.99%) [7.60]	1351 (24.13%) [3.00]

Each sub- table includes the results for the top 10 genes, when allowing the next best synonymous mutation, per TE percentage reduction constraint. For each case, in parentheses the added percentage is specified, while in square brackets the mean number of mutations performed per gene.

### Tracing the trafficking modification of the algorithm

**Figure 3:**
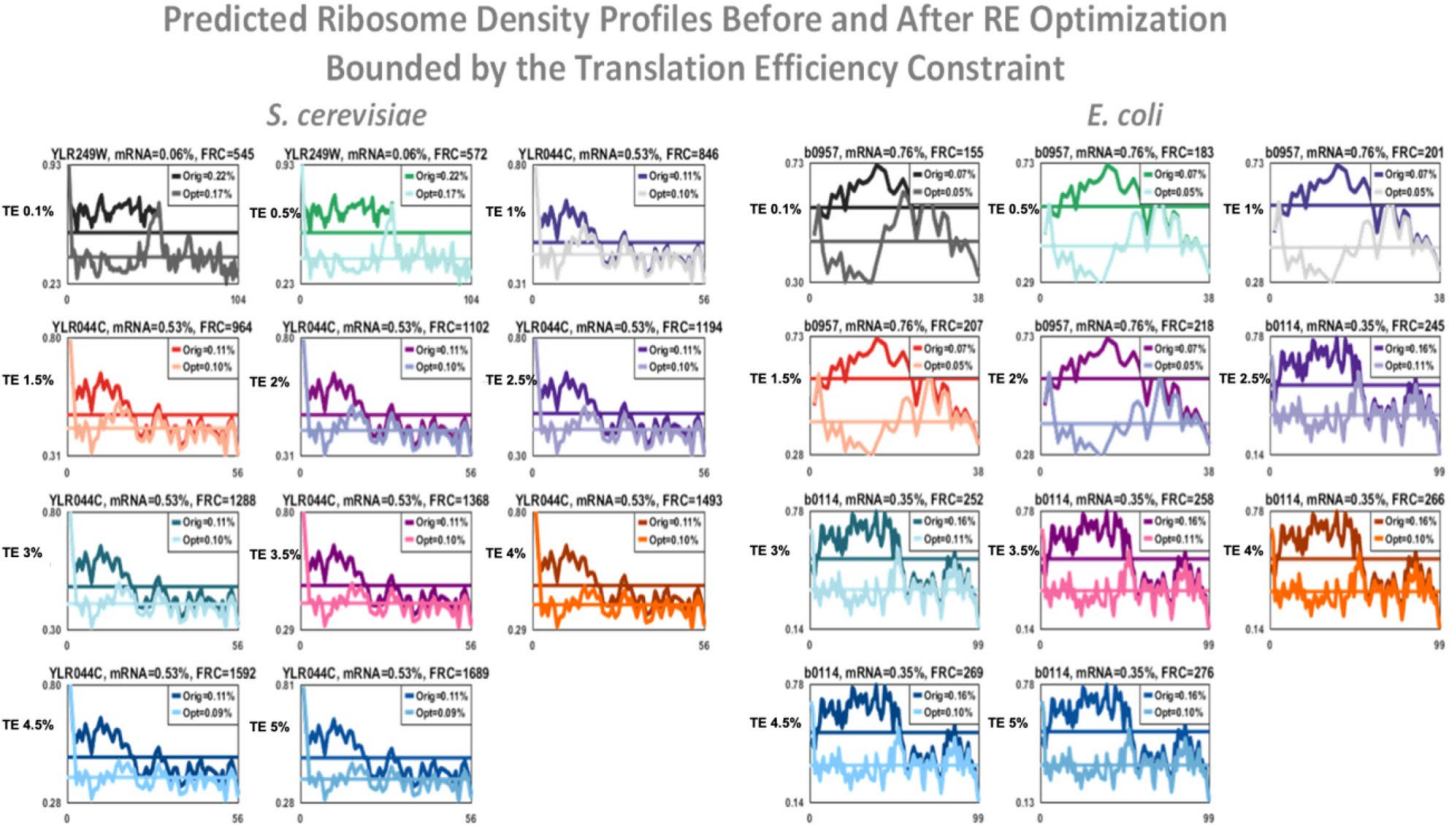
*Saccharomyces cerevisiae* and *E. coli* FGM algorithm ribosomal density profiles (10 codons resolution) for the first modified gene per translation efficiency (TE) constraint before (Orig) and after (Opt) mutations, results incorporate the effect of the first 100 mutated genes, mRNA levels as percentage of all genes is indicated, as well as each genes contribution to the free ribosome pool (FRC). You can easily see the decrease of ribosome density due to the introduced synonymous mutations. The x-axis in each sub-figure is the coordinates in the coding region of the corresponding gene at a resolution of the RFM chunks (10 codons); the y-axis is the predicted ribosomal density.

Figure 3 depicts the FGM algorithm ribosomal density (RD) profiles for *S. cerevisiae* and *E. coli* for the first modified gene per representative translation efficiency (TE) constraint (expressly 0.1%, 0.5%, 1%, 1.5%, 2%, 2.5%, 3%, 3.5%, 4%, 4.5%, and 5%) before and after mutations, results incorporate the effect of all 100 mutated genes (see Supplementary for all the *S. cerevisiae* TE groups, and the BGM and GGM variants, and all the *E. coli* profiles). As can be seen, following the algorithm the ribosomal density significantly decreases.

## Experimental results

**Figure 4:**
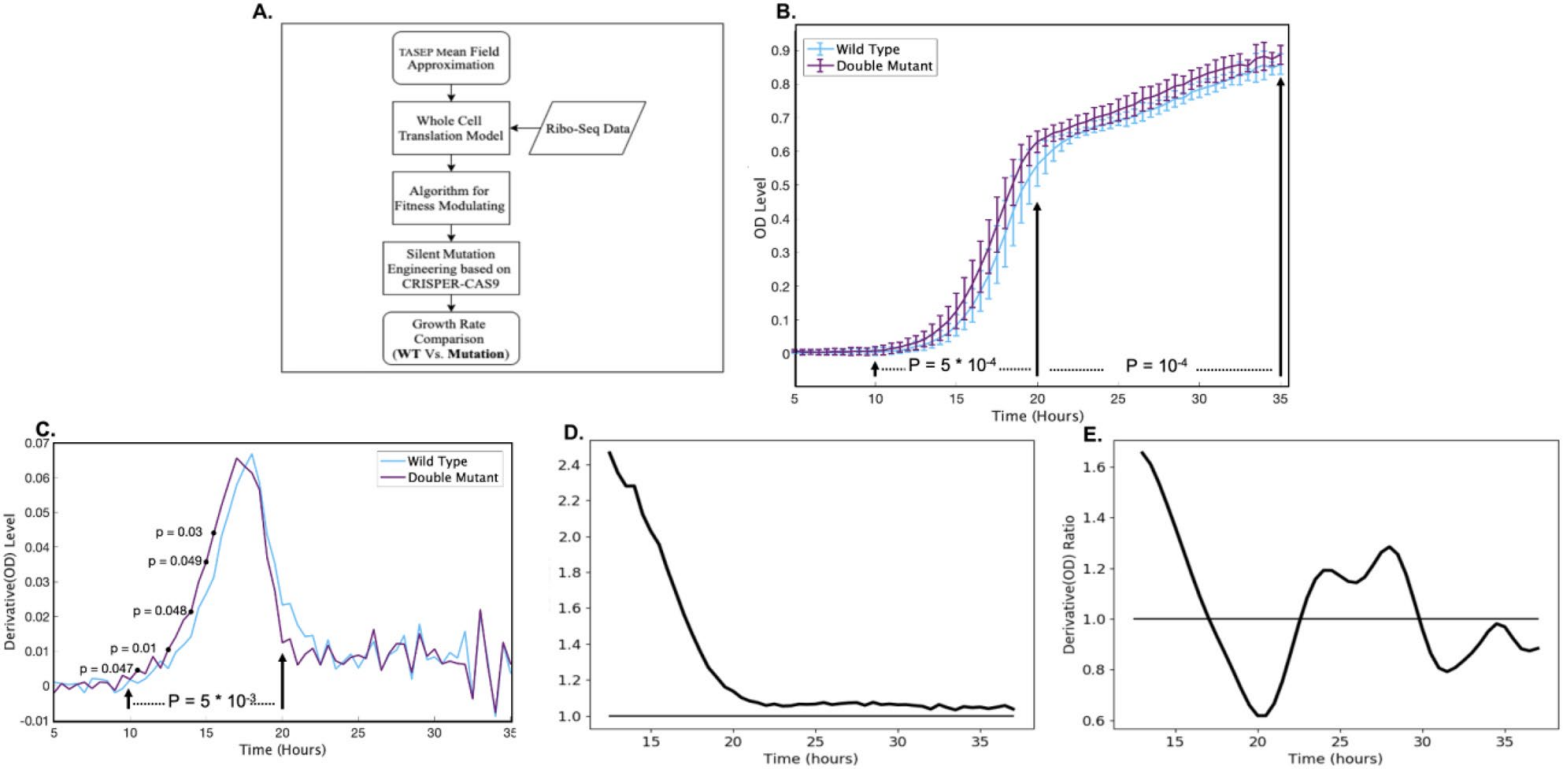
(**A**) Flow diagram of the experimental procedure. (**B**) Mean OD curve of WT and the double mutant. The titer, measured by OD, of the double mutant is higher in all time points. The bars represent the STD in each point. (**C**) Derivatives of the OD (i.e. the estimation of growth rate) of mutant and the WT; at the log phase it is significantly higher for the mutant. (**D**) The OD ratio between the mutant and the WT. The largest ratio is obtained at the exponential (log) phase. E. The estimation of the ratio in the OD derivatives between mutant and the WT. The largest ratio is obtained at the beginning of the log phase. See also supplementary figures S1–S5.

Next, we performed an experimental procedure to demonstrate our approach (flow diagram appears in Fig. 4A, and details in the Methods section). According to our algorithm, the genes RPO21 (YDL140C) and CYS4 (YGR155W) were chosen to be modified and the mutations were suggested. We then used the CRISPR-Cas9 system to introduce 8 and 7 mutations in RPO21 and CYS4, respectively, generating a double mutant strain (Fig. 4A). The mutant as well as the WT were grown in YDP and their growth rate was compared by measuring their optical density (OD) (Fig. 4A). As can be seen, the OD of the mutant is significantly higher than the WT along the growth kinetics (p-value = 10^−4^ at the stationary phase and 5*10^−4^ at the log phase via empirical permutation test; Fig. 4B) with highest ratio at the exponential growth phase, before the diauxic shift (Fig. 4D). The derivatives of the OD (i.e. the estimation of growth rate) of mutant is significantly higher than that of the WT at the log phase it (p-value 5*10^−3^ via empirical permutation test; Fig. 4C).

The highest titer increase rate (derivative; Methods) is at the beginning of the log-phase (Fig. 4E).

The results suggest that our optimization is more effective in the log phase; this may be related to the fact that our model parameters were tailored to this phase and not to the diauxic shift phase when the cell density increases, the glucose is exhausted, yeast use ethanol as a carbon source and thus stress levels increase. The results may also suggest that the relation between translation optimization and growth rate is less relevant in the diauxic shift and stationary phases as in these phases other aspects aside from translation are rate limiting.

## Discussion

In summary, we suggest an approach for improving the growth rate of cells via the via alleviating ribosome traffic jams; the approach was demonstrated experimentally on *S. cerevisiae* for improving growth rate and is expected to be useful also in the case of other organisms and viruses.

According to our analysis we may gain an increase of over 33% in the free ribosomal pool after introducing mutations to 100 genes. We'd like to emphasize the fact this can contribute to a huge increase in growth rate of cells:

It is known that translation consume most of the energy in the cell but let's assume that only 25% of this improvement will be use by the cell to improve growth rate to get 7.5% improvement. In the case of micro-organisms with doubling time of 90 min such as *S. cerevisiae* this means that in one weak we will have more than 3 × 10^3^ times more cells of the mutant in comparison to the WT. This is a huge difference for biotechnological objectives.

We believe that a similar approach to the one describe here can be used for *increasing* traffic jams and thus *decreasing* the growth rate of the target organism. This will be helpful for example, for pathogen attenuation such as viruses and bacteria for generating live attenuated vaccines.

The approach has various important advantages:

First, it is very generic as translation is a fundamental process that occurs in all cells and consumes much of the cellular energy. Thus, it can be used for all organisms across the three domains of life, as well as viruses.

Second, it introduces a minimal number of changes to the genome of the host; specifically, all the mutations are silent and we do not introduce/remove genetic material or alter the encoded proteins; unlike other previous methods^2,27,42,43^, such as gene knockouts or expanding the intracellular tRNA pool of the host by over-expressing genes encoding the rarer tRNAs, which have many caveats, most notably the disruption of the regular interplay with other cellular components^7,27,42^. For example, metabolic effects of changing the tRNA concentrations of a cell may lead to the potential induction of an immune response in vertebrates as a result of under-acetylated tRNA^42^.

Third, it enables generating a growth rate gradient based on the number of introduced mutations, which usually have an additive affect. Thus, it should enable obtaining the exact required growth rate, which has significant economic implications.

Fourth, it is based on a computational model (and not trial & error) making it more predictable, flexible and cost-effective.

Finally, since our approach is based only on silent mutations and aiming at not dramatically affecting the translation rates of specific mRNAs it can easily be integrated with other (more traditional) approaches such as the optimizing of only the heterologous gene or performed metabolic engineering which includes the introduction of non-silent mutations. For example, if we want to improve the yield of the production of a certain heterologous protein it will be a good idea to improve both the growth rate of the host and global ribosomal allocation (our algorithm) and to optimize the sequence of the heterologous gene for optimal expression. In many cases, any addition improvement in the yield can have significant impact on the cost of the product and its economical feasibility.

The approach can also be utilized for other/diverse biotechnological goals. As mentioned, it can be used for improving the efficiency of heterologous gene expression by improving the ribosomal allocation in endogenous and heterologous genes. However, it can also be used for other objectives: for efficient production of inactivated vaccines via improving the growth rate of the pathogen before killing it; for improving the growth rate of various crops in agriculture, and for engineering the bacterial composition of the microbiome.

It is important to mention that the suggested approach may be extended to other cellular processes/resources such as freeing RNAPs traffic jams, amongst others. Here we focused on translation since this is the process with the best whole cell computational models, which consumes most of the intracellular energy.

Interestingly, the results reported here may suggest that translation, though extensively optimized by evolution, can be further significantly augmented. We are able to further optimize endogenous genomes since during the process of evolution mutations occur randomly and are fixed with a probability increasing with their effect on fitness.

On the other hand, our approach is based on finding optimal *sets* of mutations such that each of the mutations may have a small effect (with a small fixation probability), but jointly they have a very strong effect. Thus, these solutions "can be found" by evolution only with a very small probability, while we are able to identify them via our approach easily.

Hence, this line of research also explores the ability of evolution to solve biophysical problems such as ribosome allocation.

Finally, the experiments reported here indeed suggest that the decrease in traffic jams is related to the increase in growth rate. This may be related to the increase in the free ribosome pool that has a positive global effect on translation and thus on growth. This can also be additionally possibly related to the fact that the energy freed due to the deletion of some traffic jams can be used in other intracellular processes. Indeed, previous studies have suggested that growth rate is related to increasing the free ribosomal pool^44–49^ .

We cannot completely rule out the possibility that the genes we edit here (e.g. RPO21) contribute to the growth rate due to a potential change in their activity. However, we believe that this is a less probable explanation since our mutations did not affect the encoded proteins and aimed at maintaining the translation rate of the mRNA (i.e. keeping its relative protein levels similar to the WT).

In the future is will be interesting to further explore the research reported here in various directions: First, it will be interesting to repeat the reported experiment on many additional genes and their combinations. Second, it will be interesting to measure the effect on the translation rate of the mutated genes; this is challenging since today there is no simple technique for measuring small changes (a few %) in the translation rate of endogenous genes. Third, it will be interesting to implement it on additional organisms.

## Methods

### Ribosomal Profiling Data

*Saccharomyces cerevisiae* ribosomal profiling raw reads (2 replicates) and mRNA levels (2 replicates) were taken from^50^. *E. coli* ribosomal profiling raw reads (1 replicate) and mRNA levels (1 replicate) were taken from^51^.

### Reference *S. cerevisiae* and *E. coli* Genome Assemblies

*Saccharomyces cerevisiae* genomic data (R64-1-1) was downloaded from BioMart^52^. 5′UTR and 3′UTR annotations (spliced) were obtained from^53^. We compiled what we call a reference ‘genome’ by taking unspliced transcripts (in this case unspliced ORFs) and flanking them with upstream and downstream segments up to 1000nt, with the constraint they cannot overlap annotated ORFs unless this causes the segment to be under 30nt (approximate ribosomal footprint^12^). A reference transcriptome was compiled similarly, only with annotated ORFs, annotated UTRs were added to the ORFs when available, otherwise flanking segments were supplemented as described. Since there is no alternative splicing in *S. cerevisiae*, both the genome and transcriptome contain 6664 genes. There were 4415/6664 annotated 5′UTRs, and 5126/6664 3′UTRs. Considerable specific rRNA contamination may remain even after depletion by subtractive hybridization. Thus, a significant fraction of sequencing reads are derived from digested rRNA present in the monosome sample. Therefore reads mapping to rRNA are first filtered, against a rigorous rRNA database^12^. Aside from rRNA contamination, there are contaminating sequences derived from other abundant ncRNAs, such as tRNAs. The extent of rRNA and ncRNA contamination can vary, particularly when global changes in protein synthesis alter the fraction of active ribosomes, and thus the number of ribosome-protected footprints relative to other RNAs. Thus, reads are also mapped separately to an annotated non-coding RNA database^12^. rRNA (16 genes), tRNA (299 genes), ncRNA (15 genes), snRNA (6 genes) and snoRNA (77 genes) databases were compiled from BioMart (sc_R64-1-1)^52^.

*Escherichia coli* genomic data for strain k-12 MG1655 (ASM584v2.31) was downloaded from Ensembl Bacteria^54^. The genome and transcriptome was compiled similarly to *S. cerevisiae*. No annotated UTRs were available, and since the *E. coli* genome is compact we substituted flanking segments of 200nt instead of 1000nt, and ensured that also these pseudo UTRs were non-overlapping with annotated ORFs, again unless this causes the segment to be under 27nt (approximate ribosome size^55^).The *E. coli* genome has 4140 protein coding genes, 22 rRNAs, 86 tRNAs, and 65 ncRNAs.

### Mapping ribosomal footprints and mRNA fragments

The following read (ribosomal footprint or mRNA fragment) mapping protocol was devised and implemented, for each of the replicates separately:

1. The 3′end adapter CTGTAGGCACCATCAAT for *S. cerevisiae* was removed from the 51nt long reads using Cutadapt v1.6^56^, retaining only reads with a minimum length of 24nt and maximal length of 34 for ribosomal footprints, and 24-40nt for mRNA fragments. For *E. coli* read lengths of 20-42nt were retained according to^51^.
2. These reads were then initially mapped against the respective non-coding databases, using Bowtie v1.1.2^57^: -a –best –strata -n 2 –seedlen 21 –tryhard. For *E. coli* a seed length of 20 was used. In -n mode, alignments may have no more than N mismatches in the seed, which was chosen here to be 2^13^, with the seed length being 21, as sequencing errors are more likely near the end of the read. Specifying -a instructs bowtie to report all valid alignments, subject to the alignment policy, enabling us to control the mapping selection process, with –best –strata causing bowtie to report only those alignments in the best alignment "stratum". Throughout the analysis the Bowtie mapping is executed as described. Reads which mapped against the non-coding databases were removed.
3. The remaining reads were first mapped against the assembled ‘genome’ using Bowtie as described. The read mapped position is at first attributed to the read’s 5′ end first nucleotide (Bowtie default), and is then determined according to the heuristic below. Uniquely mapped reads are identified accordingly. As discussed in^58^, many of the multi-aligned reads are attributable to known duplicated genes and segmental duplications. This is expected for paralogs that are very similar to each other and for internally repeated domains within some genes. If all multi-aligned reads are simply discarded, the end result will be to undercount greatly or even entirely fail to report expression for genes that have closely related paralogs, such as those of the ubiquitin family for example. Specifically, in our dataset, the human transcriptome, many of the alternatively spliced transcripts of a gene bear high similarity. Multiple aligned reads were extended to 30/27nt for *S. cerevisiae* and *E. coli* respectively (the respective approximated ribosome size), with a mismatch score calculated. Reads with a single minimal mismatch score were deemed unique. Multi-aligned reads were handled after the A-site shift was determined for ribosomal footprints (mRNA fragments mapped position is assumed to be the 5′end first nucleotide). We calculate the A-site shift as a function of the read lengths (a range of 24-34nt and 20-42nt, for *S. cerevisiae* and *E. coli* respectively, as determined by Cutadapt) at the start codon of the uniquely mapped reads, guided by the logic that the offset between the ribosome A-site and the start of the footprint would be of different proportion in the varying read lengths^59,60^. We look at reads mapped in the vicinity ± 50nt of the start codon, and define the ribosomes real A-site to be 15nt^12^ and 12nt for *S. cerevisiae* and *E. coli* respectively, we then heuristically hypothesize the read length adjusted A-site position according to the following formula:
  $$\begin{aligned} {\text{ASShift }} & = {\text{ realAS - round}}\left( {\left( {\text{riboSize - readLength}} \right){/2}} \right){\text{; if the read length is shorter than the ribosome size}} \\ {\text{ASShift }} & = {\text{ realAS + round}}\left( {\left( {\text{readLength - riboSize}} \right){/2}} \right){\text{; otherwise}} \\ \end{aligned}$$
  Where ASShift is the resultant hypothesised A-site shift, realAS as defined is 15/12nt, riboSize was taken to be 30/27nt, for *S. cerevisiae* and *E. coli* respectively, and readLength is the read length as determined by Cutadapt. We used Matlab’s findpeaks function to find local maxima in the profile induced by the respective read length group mapping. We sorted the local peaks according to prominence and then tested the top three, with the one closest to our hypothesized A-site shift being selected.
  Multi-aligned reads were first tested to see if they overlap annotated ORFs, if so they were removed from the multi-aligned contenders (in a few instances this resulted in a uniquely mapped read). Equal contenders vicinity read density was calculated 30/27nt, for *S. cerevisiae* and *E. coli* respectively, upstream and downstream of the mapped read’s A-site (the read mapped position). Each of the multiple mapped positions is then assigned a fraction of the read, signifying its relative frequency based on its vicinity read density. In some rare instances the vicinity read density of all the multi-aligned reads is zero (possibly reflecting very recent gene duplication^58^), we then distribute the reads evenly among the mapped positions candidates. The inclusion and proportionate distribution of multiple aligned reads will naturally have variable impact on RNA quantification, with smaller effects on paralogs that are more divergent and larger effects on those that are more similar to each other ^58^.
4. Unmapped reads were then mapped to the transcriptome to account for splice junctions.
5. Reads mapped to the transcriptome are integrated into the genome mapping according to the exon positions. Total read count per gene is then calculated according to exon mappings only, with the respective ribosome footprint size taken from the UTRs.

### Whole cell simulation to infer RFMNP parameters

We used the RFMNP (RFM (Ribosome Flow Model) network with a pool)^28^ to model translation, which is a general dynamical model for large-scale simultaneous mRNA translation and competition for ribosomes based on combining several ribosome flow models (RFMs)^41^, each representing a single copy of a gene, interconnected via a pool of free ribosomes.

According to the RFM a ribosome that occupies the *i-th* site moves, with rate $\lambda_{i}$, to the consecutive site provided the latter is not occupied by another ribosome. Denoting the probability that the *i-th* site is occupied at time *t* by $p_{i} \left( t \right)$, it follows that the rate of ribosome flow into/out of the system is given by: $\lambda \left[ {1 - p_{1} \left( t \right)} \right]$ and $\lambda_{n} p_{n} \left( t \right)$ respectively. Hence, the rate of ribosome flow from site *i* to site *i* + *1* is given by: $\lambda_{i} p_{i} \left( t \right)\left[ {1 - p_{i + 1} \left( t \right)} \right]$. Thus we get the following set of differential equations that describe the process of translation elongation:

$$\left\{ {\begin{array}{*{20}c} {\frac{{dp_{1} (t)}}{dt} = \lambda \left[ {1 - p_{1} (t)} \right] - \lambda_{1} p_{1} (t)\left[ {1 - p_{2} (t)} \right]\,\,\,\,\,\,\,\,\,\,\,\,\,\,\,\,\,\,\,\,} & {} \\ {\frac{{dp_{i} (t)}}{dt} = \lambda_{i - 1} p_{i - 1} (t)\left[ {1 - p_{i} (t)} \right] - \lambda_{i} p_{i} (t)\left[ {1 - p_{i + 1} (t)} \right]} & {1 < i < n} \\ {\frac{{dp_{n} (t)}}{dt} = \lambda_{n - 1} p_{n - 1} (t)\left[ {1 - p_{n} (t)} \right] - \lambda_{n} p_{n} (t)\,\,\,\,\,\,\,\,\,\,\,\,\,\,\,\,\,\,\,\,\,\,} & {} \\ \end{array} } \right.$$

The interconnection between the single RFMs, which are now extended single-input single-output control system, is performed via the initiation rate of each RFM (gene), which is the input to the system, modeled as: $G_{j} = \lambda_{0j} {\text{tanh}}\left( {Z/c} \right)$, where $\lambda_{0j}$ denotes the initiation rate of gene *j*, *Z* denotes the free pool of ribosomes, and *c* is a parameter of the model fitting local and global aspects of the free ribosomal pool. The use of tanh is appropriate for modelling a saturating function, and is a standard function in ASEP (asymmetric exclusion processes) models with a pool, because it is 0 when Z is 0, uniformly bounded and strictly increasing for Z ≥ 0. Furthermore, for Z ≤ 0 the function tanh(Z) takes values in [0,1) so it can also be interpreted as a probability function. The translation rate becomes the output of the system.

The steady state translation rate *R* is calculated as follows: briefly (for full details see^41^), in steady state the occupation probabilities are constant in time and equal to $\left\{ {\pi_{1} , \ldots ,\pi_{n} } \right\}$, thus

1 $$R = \lambda_{n} \pi_{n}$$

This rate is also equal to the steady state rate at which ribosomes leave the mRNA strand (after translating the entire sequence). At steady state the left hand side of Eq. (1) vanishes and we get:

2 $$\left\{ {\begin{array}{*{20}l} {\lambda \left[ {1 - \pi_{1} } \right] = \lambda_{1} \pi_{1} \left[ {1 - \pi_{2} } \right] = R} \\ {\lambda_{i - 1} \pi_{i - 1} \left[ {1 - \pi_{i} } \right] = \lambda_{i} \pi_{i} \left[ {1 - \pi_{i + 1} } \right] = R 1 < i < n} \\ {\lambda_{n - 1} \pi_{n - 1} \left[ {1 - \pi_{n} } \right] = \lambda_{n} \pi_{n} = R} \\ \end{array} } \right.$$

Solving Eq. (2) for *R* can be done numerically. We performed an iterative implementation of RFMNP, due to the intense time and memory requirements of solving the entire ODE system.

The RFMNP has three parameters which need to be estimated, initiation rates, codon elongation rates, and *c*. We developed a novel iterative algorithm for this purpose:

Initial initiation rates were estimated to be the measured ribosomal read count divided by the mRNA levels (Ribo-Seq measurements described above) and then normalized to have the median of the estimated median initiation rate which is 0.8 per second for *S. cerevisiae*^61^ and 0.6 for *E. coli*^62,63^.

Initial codon elongation rates were calculated based on the tRNA Adaptation Index (tAI^64^) as described in^65^ (see RFMapp User Guide in http://www.cs.tau.ac.il/~tamirtul/RFM_Installers/install.htm) with a minor adjustment. The logic is that elongation rates are determined by the codon composition of each site and the tRNA pool of the organism. Briefly, the elongation rate associated with a codon is proportional to the abundance of the tRNA species that recognize it, taking into account the affinity of the interactions between the tRNA species and the codons.

Let *n*_*i*_ be the number of tRNA isoacceptors recognizing codon *i*. Let *tCGNij* be the copy number of the *j*th tRNA that recognizes the *i*th codon, and let *S*_*ij*_ be the selective constraint on the efficiency of the codon-anticodon coupling. We define the *absolute adaptiveness*, *W*_*i*_, for each codon *i* as:

$$W_{i} = \sum\limits_{j = 1}^{{n_{i} }} {(1 - S_{ij} )tCGN_{ij} }$$

The *S*_*ij*_-values can be organized in a vector (*S*-vector) as described in^64^; each component in this vector is related to one wobble nucleoside-nucleoside paring: I:U, G:U, G:C, I:C, U:A, I:A, etc. Eukaryotic and prokaryotic *S* values were taken from^66^.

From *W*_*i*_ we obtain *p*_*i*_, which is the probability that a tRNA will be coupled to the codon:

$$p_{i} = \frac{{W_{i} }}{{\sum\nolimits_{j = 1}^{61} {tCGN_{j} } }}$$.

We normalize $p_{i}$ to have the median of the estimated codon rate which is 6.4 aa/s (growth rate range 2.8–10.0)^67^ in *S. cerevisiae*, and 13.5 aa/s (growth rate range 5–22)^62,68,69^ in *E. coli*. Also in *S. cerevisiae* the CGA codon according to tAI is disproportionally slow, and thus we set it to be 10 times the slowest codon. The expected time on codon *i* $t_{i} = 1/p_{i}$*.* We coarse grain each gene into sites/chunks of *C* codons as described in^65^, thus for each chunk the codon times are summed, and the chunk rate is: $1/\sum\nolimits_{i = 1}^{C} {t_{i} }$. We used a chunk size of 10 codons (the approximate size of the ribosome^70^) for *S. cerevisiae* and 9 codons for *E. coli*^55^. If the last chunk is 5 codons or less, it is incorporated in the chunk before it, in-order to avoid extremely fast chunks which would distort the simulation.

**Figure 5:**
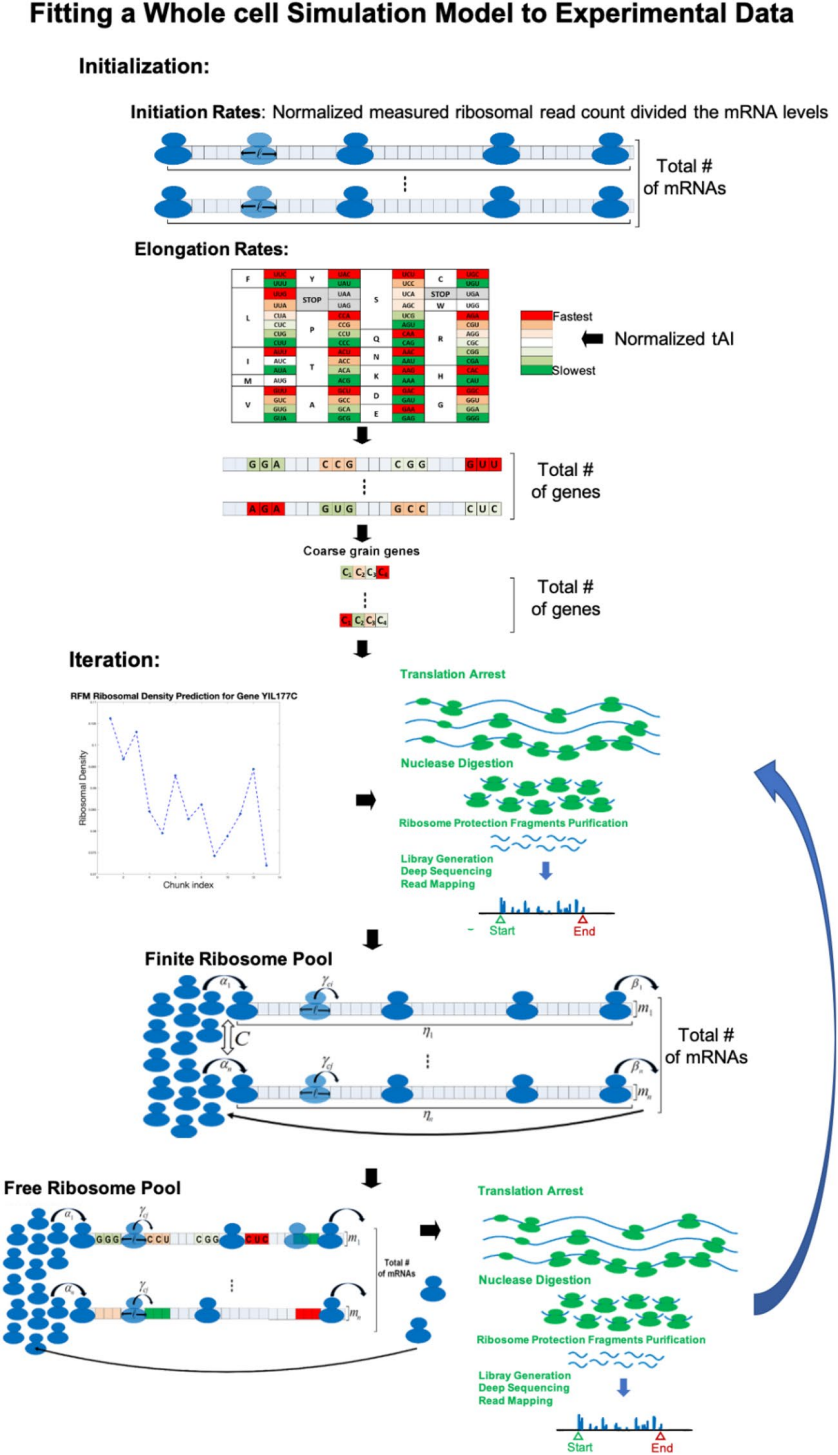
An illustration of our parameter estimation model. The estimation procedure iterates between three major sub-steps (upper to lower parts of the figure): (1) Optimize codon decoding rates that optimizes the correlation between the model prediction of ribosomal density and the experimental measurements based on ribo-seq while maintaining the correlation with tAI, (2) Estimation of local translation initiation rate codon decoding rates that optimizes the correlation between the model prediction of ribosomal density and the experimental measurements based on ribo-seq, (3) Estimation of the size of free pool of ribosome that induces consistency in terms of the total number of ribosomes in the cell and the ribosomal densities on the mRNAs. See more details in the text above.

We then perform the following iterative steps (Fig. 5):

1. The initiation rates are optimized for each gene separately utilizing the RFM, via simulated annealing by increasing or decreasing the current initiation rate by 5% until $abs\left( {rd_{j} - rcm_{j} } \right) < \varepsilon$, where $rd_{j}$ is the estimated RFM ribosomal density for gene *j*, $rcm_{j}$ is ribosomal read count divided by the mRNA levels of gene *j*, and $\varepsilon$ is 10^−3^. Instead of having the initiation rate as a separate parameter/chunk in the RFM calculation, we incorporate it into the first chunk so that a more balanced estimation of the initiation rates is possible, as when simulated as a standalone chunk the initiation rate is estimated to be disproportionally high. In order for the ribosomal read count divided by the mRNA levels (*rcm*) to be on the same scale of the predicted RFM ribosomal density, we normalized it to have the median of the median ribosomal coverage of an mRNA molecule from^71^ for *S. cerevisiae* and^72^ for *E. coli* (see exact numbers below).
2. Utilizing the optimized initiation rates, and the initial elongation rates, we performed an iterative implementation of RFMNP in order to estimate *c*, and instead of solving an ODE system as originally suggested per gene^41^, we utilize a novel, linear-algebraic approach linking the protein translation rate to the maximum eigenvalue of a symmetric, non-negative tridiagonal matrix whose components are functions of the initiation and elongation rates^73^, which provides a substantial speedup, the translation rate which is what we use as a proxy of translation efficiency (TE) is the square root of the maximal eigenvalue:
  Let us denote *H* as the number of ribosomes in the system, *Z* as the number of free ribosomes, (*H* and *Z* are determined according to the literature, see below), $M_{j}$ the number of mRNA copies of gene *j*, and $x_{i}^{j}$ the number of ribosomes on segment/chunk *i* in gene *j*. In steady-state: $H = Z + \left( {\mathop \sum \limits_{j} \mathop \sum \limits_{i} x_{i}^{j} \cdot M_{j} } \right)$.
  First iteration: Begin by guessing $c_{0}$, since the logical range for *c* is between the smallest positive floating-point number and *H* we chose $c_{0} = median\left( {\left[ {s,H} \right]} \right)$, which determines the initiation rate: for each gene *j*, the initiation rate $G_{0j} = \lambda_{0j} {\text{tanh}}\left( {Z/c_{0} } \right),$ where $\lambda_{0j}$ is the estimated local initiation rate, and ${\text{tanh}}\left( {Z/c_{0} } \right)$ the global initiation rate of gene *j*, and the RFM model for every gene separately until convergence: $Z_{1} = H - \left( {\sum\nolimits_{j} {\sum\nolimits_{i} {x_{i}^{j} } } \left( 0 \right) \cdot M_{j} } \right)$.
  *K*th iteration: $Z_{k} = H - \left( {\sum\nolimits_{j} {\sum\nolimits_{i} {x_{i}^{j} } } \left( {k - 1} \right) \cdot M_{j} } \right)$, with *k*-1 being the resultant density of the previous iteration as input to the current. A binary search is performed on the *c* range and
  $$G_{kj} = \lambda_{0j} {\text{tanh}}\left( {Z/c_{k} } \right)$$
  Termination condition: $abs\left( {Z - Z_{k} } \right) < \varepsilon$, with $\varepsilon$ being 10^2^.
3. We greedily optimize the codon elongation rates to maximize the correlation between measured (Ribo-Seq) ribosomal density (RD) and predicted RFMNP RD (by concatenating the measured and RFMNP RD profile of each gene into one vector respectively, taking into account mRNA levels). In each iteration we iterate the 61 codons according to four order schemes: i. From slowest to fastest. ii. From fastest to slowest. iii. From most frequent to least frequent. iv. For 100 random permutations of the order (which are predefined and the same 100 random permutations are utilized throughout the algorithm iterations). First, calculate the initial correlation between Ribo-Seq and RFMNP RD (which is 0.7, p < 10^−308^). Then, for each of the order schemes we iterate the codons, and for each codon we test if reducing/increasing its translation time by a specified percentage epsilon improves the correlation, while constraining the new codon times to have at least a 0.5 Pearson and Spearman correlation with the original tAI estimated codon rates. Finally, the most successful scheme is selected from the 103 orders, and this determines the optimized codon elongation rates for the next iteration. We tried this with epsilon being 1%, 5%, 10%, 15%, 20%, 25%, 30%, 35%, 40%, 45%, 50%, 55%, 60%, 65%, 70%, 75%, 80%. The correlation with Ribo-Seq were robust across the percentage groups ranging from 0.74 – 0.85, and 0.82 – 0.85, in *S. cerevisiae* and *E. coli* respectively. The estimated codon elongation rates resulting from epsilon being 50% and 35% were selected for *S. cerevisiae* and *E. coli* respectively, though results are robust across the percentage groups (see Supplementary Methods for further details).

The 3 algorithm steps are performed iteratively (with the initiation rates recalculated with the new optimized codon elongation rates, and *c* estimated utilizing the newly optimized initiation rates and codon elongation rates) until no improvement larger than 10^−4^ on the 3^rd^ step’s correlation can be made.

The number of *S. cerevisiae* ribosomes used in the simulation was 200,000^74^, with 60,000 mRNAs^75^, scaled according to the mRNA levels calculated. According to^71,75^ the number of free ribosomes in the pool is ~ 15%, thus 30,000. The median ribosomal coverage is 0.1322^71^.

The number of *E. coli* ribosomes used in the simulation was 40,000 (growth rate range of 6800–72,000^72^), with 4400 mRNAs (growth rate range of 1000–7800^69,76^). The average length of the transcript portion encoding a gene is 1000nt^77–80^, was used to calculate the number of mRNAs in the cell from^69^, and the median ribosomal coverage which is 0.3105 (based on a 60nt average distance between ribosomes^72^, 27nt ribosome size, and average mRNA length).

Results are robust to variations in the selected parameters.

The code of the model for academic usage appears in the supplementary file code.zip.

### Parameter estimation randomization tests

**Figure 6:**
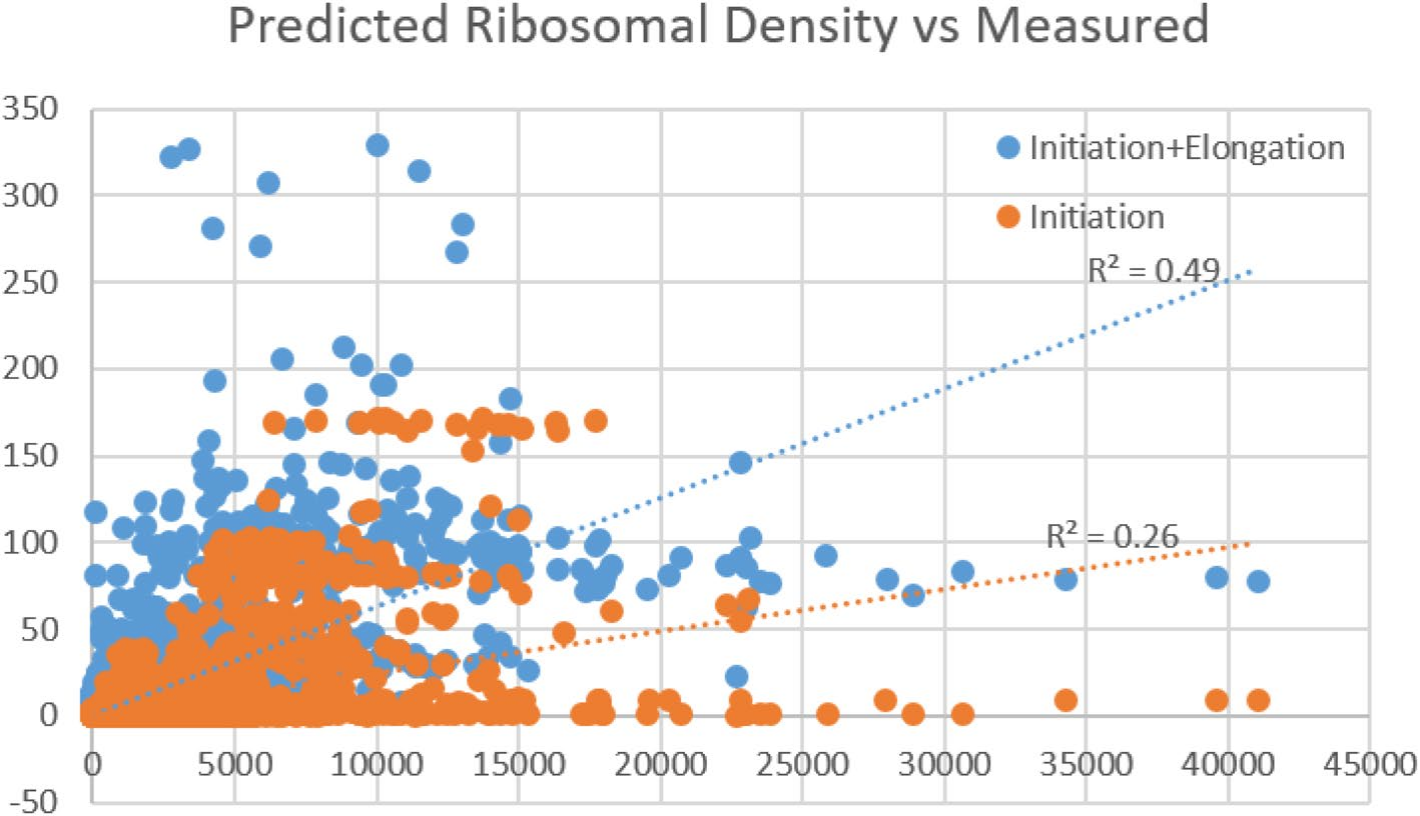
Results of the *S. cerevisiae* randomization test. The x-axis depicts the measured ribosomal density (Ribo-Seq), while the y-axis depicts our model prediction, showing we can explain 49% of the variability of the measured data. The 100 random models achieved a mean correlation of 0.51, thus we can deduce that the initiation explains 26% of variability of the measured data, and elongation 23%.

To show that the correlation achieved between Ribo-Seq and RFMNP RD in the previous section is indeed related to the elongation rates (i.e. the initial tAI estimation values and the subsequent optimization), and simultaneously estimate what fraction of the ribosome density variability can be explained by initiation, we performed the following 100 randomizations. We randomly permuted the tAI predicted codon times and calculated the codon elongation rates according to those randomized times, and then performed step 1 and step 2 of our estimation algorithm once. We then calculated the predicted RFMNP RD for each of the randomizations and correlated it with the Ribo-Seq RD as described above. For *S. cerevisiae* the real correlation achieved for the first iteration was r^2^ = 0.49 (r = 0.70, p < 10^−308^), while all 100 randomizations achieved a lower correlation with a mean value of r^2^ = 0.26 (r = 0.51), giving an empirical p-value of 0. Similar results were achieved for *E. coli*, where the real correlation achieved for the first iteration was r^2^ = 0.67 (r = 0.82, p < 10^−308^), while all 100 randomizations achieved a lower correlation with a mean value of r^2^ = 0.59 (r = 0.77), giving an empirical p-value of 0. This result is strong as we optimized the initiation rates according to the randomized elongation rates and real Ribo-Seq measurements, thus coupling the initiation and elongation in a synergistic manner. See Fig. 6 below.

We performed another significance test, where we used the first iteration optimized initiation rates and *c*, and then predicted the size of the free ribosomal pool while permuting the codon elongation rates based on the initial codon rate tAI estimation values (i.e. unoptimized codon rates) 100 times (in the same manner as the test above). In all cases the free ribosomal pool was lower than the real *S. cerevisiae* free ribosomal pool of 30,000 ribosomes, giving an empirical p-value < 0.01, with the mean predicted free ribosomal pool being 9421. Similar results were achieved for *E. coli* where in all cases the randomized free ribosomal pool prediction was lower than the *E. coli* free ribosomal pool of 5600 ribosomes, giving an empirical p-value < 0.01, with the mean predicted free ribosomal pool being 2630.

### Whole cell simulation of traffic jam engineering

We used the inferred initiation rates, codon elongation rates, and *c*, in order to determine the optimal ramp mutations across the host genome, according to the following TJE (Traffic Jam Engineering) greedy algorithm. A mutation is defined as a gene location, we define a location as the first nucleotide (nt) of a codon, for example, if the second codon is mutated, its location within a gene would be the fourth nt. An RE step is defined as:

Iterate all the host genes, for each gene we look at the first 50 codons (disregarding the first 10 codons due to important regulatory signals^37^), and mutate a codon to its slowest synonymous codon, as long as it does not reduce the gene’s translation rate (as calculated by the RFMNP) beyond some threshold $\tau$, we chose 0.1%, 0.5%, 1%, 1.5%, 2%, 2.5%, 3%, 3.5%, 4%, 4.5%, and 5% as thresholds. The best mutation is the one that most increases the free ribosome pool, and it is selected. We would like to emphasize the fact that increasing $\tau$ is expected to improve the performances of the algorithm in terms of the increase in the free pool of ribosomes (see supplementary figure S6. However, our aim of using a threshold $\tau$ is to change only the ribosomal traffic jams without affecting other aspects that we do not know how to model and/or that are not included in our model. One such aspect is the encoded amino acid sequence of the proteins. A second aspect was the relative translation rate (and thus protein levels) of different proteins; we believe that extreme changes in the protein levels of some proteins can be deleterious to the cell. Thus, we wanted the relative change in the translation rate to be close to zero and thus $\tau$ = 5% was the highest value we allowed in the paper.

Iterating one mutation at a time across the entire genome is overly time consuming, and also counterproductive, as ultimately we would like to minimize the number of genes we are mutating, due to experimental constraints. Thus we developed 3 variants of the above approach which operate at the gene level:

1. Forward Gene Minimization (FGM): Per gene start at the beginning of the ORF and incorporate all mutations that improve the free ribosomal pool while not reducing the gene’s translation rate beyond some threshold $\tau$. In each iteration the gene which most increases the free ribosomal pool is selected.
2. Backward Gene Minimization (BGM): Similarly to FGM only now we start at the end of the gene’s ORF and traverse backwards. The logic for this variation is that since many important signals are encoded at the beginning of the ORF^37^ we may want to maintain them.
3. Greedy Gene Minimization (GGM): Per gene iterate over all possible mutations and choose the one which most increases the free ribosomal pool. Repeat this procedure until no more mutations can be selected without violating the translation rate threshold $\tau$. Select the gene which most increases the free ribosomal pool.

One could continue until there is no improvement, however we decided to terminate after the best 100 genes were selected as practically/currently we will not introduce more mutations to generate novel engineered genomes.

This approach allows a gene to be mutated only to its free ribosomal pool improving slowest codons, but next best slowest codon can also potentially improve fitness. There are of course many variants of this scheme, we allowed the next best slowest codon to be selected if the slowest one could not be selected due to TE constraint breach, and it did further increase the free ribosomal pool. All codons slower than the original are tested in descending order until one matching the criteria is found.

## Experimental details

### Generating synonymous mutants

The CRISPR system was used to mutate the top selected genes in *S. cerevisiae* BY4741 (MATa). Guide RNA (gRNA) and donor DNA sequences were designed, and the relevant sequences were generated. To generate the gene-specific gRNA, plasmid pNA0525 with LEU2 marker was linearized with NotI. Gibson assembly protocol was used to clone the gene-specific gRNAs into pNA0525, where it was fused to structural crRNA and both were expressed from a SNR52 promoter. Cells were grown to mid-log and transformed first with plasmid pNA0519, which contains the cas9 gene (expressed from a TEF promoter) and a HIS3 marker. Transformants were selected on histidine drop-out plates. Cas9 expressing cells were grown to mid-log and transformed with the relevant gRNA plasmid and gene-specific donor DNA (which contains the synonymous mutations that we to introduced to the specific gene, using homologous recombination). Transformants were selected on both histidine and leucine drop-out plates. Candidates were checked by PCR with primers that span the mutations region, followed by sequencing. Synonymous positive clones were isolated and kept for analysis. We studied three mutants: RPO21, CYS4, RPO21 + CYS4 (double mutant that include the mutations in the two genes: RPO21 and CYS4).

All mutants were grown in YPD following their final analysis, and the loss of their plasmids was verified, including the WT control which were BY4741 cells transformed with pNA0519 and then made lose the plasmid. The growth kinetics of all the mutants mentioned above was examined. As can be seen, the supplementary figures S2–S5 both mutants exhibit higher titer and growth rate than the WT; the results of the double mutant (mentioned in the main text) where more statistically significant.

The original and mutant sequences for the two genes are as follows:

**YGR155W|CYS4, Original ORF (first 135 bp):**

ATGACTAAATCTGAGCAGCAAGCCGATTCAAGACATAACGTTATCGACTTAGTTGGTAACACCCCATTGATCGCACTGAAAAAATTGCCTAAGGCTTTGGGTATCAAACCACAAATTTATGCTAAGCTGGAACTA.

**Mutated ORF (7 mutations):**

ATGACTAAATCTGAGCAGCAAGCCGATTCACGGCATAACGTTATAGACTTAGTTGGGAACACGCCGTTGATCGCTCTGAAAAAATTGCCTAAGGCTTTGGGTATCAAACCACAAATTTATGCTAAGCTGGAGCTA.

**YDL140C|RPO21, Original ORF (first 134 bp):**

ATGGTAGGACAACAGTATTCTAGTGCTCCACTCCGTACAGTAAAAGAGGTCCAATTCGGTCTTTTCTCACCTGAAGAAGTTAGAGCAATCAGTGTGGCCGCCAAAATTAGATTTCCAGAGACAATGGATGAAAC.

**Mutated ORF (8 mutations):**

ATGGTAGGACAACAGTATTCTAGTGCTCCACTCCGAACAGTAAAAGAGGTTCAATTCGGGCTTTTCTCACCTGAGGAAGTTCGTGCAATAAGTGTGGCAGCAAAAATTAGATTTCCAGAGACAATGGATGAAAC.

### Growth experiments

Single cells of synonymous mutants along with WT cells were suspended in PBS buffer and counted using Scepter (Millipore) or a microscope. 4000 cells were inoculated into 110 μl YPD in 96 well plates and grown at 30 degrees, shaking at 280 rpm. Growth kinetics were measured in a Tecan spectrophotometer every 30 min until cells reached stationary phase. Growth of mutants was compared to that of the WT control by plotting the OD as a function of time for each of the strains. The mean STD across biological replications is only 10% of the mean signal which is very typical for this type of experiments even in flasks.

### Analysis of the experimental results

For each of the 7 biological repeats of the mutant and WT, OD levels of each of the wells were aligned to the beginning of their log phase. Significance of the pattern was estimated by 10,000 randomizations as follows: For each of the biological repeats we permuted a random number of wells between the WT and DM; then we averaged each permuted type (WT and DM) over all the repeats and in some cases also over a certain range (e.g. entire stationary phase or entire log phase). The number of times the difference of these averages between the permuted DM and WT is higher than the original difference between the two values out of the 10,000 randomization was used as an empirical estimation of a p-value.

All biological repeats that resulted with DM OD levels higher than WT OD levels in the stationary phase (5 out of 7 repeats) are significant (p-value ≤ 0.05). The general p-value of the averaged repeats is p = 10^−4^ for the entire stationary phase. We also reported the OD ratio between the mutant and the WT, and the ratios between the derivatives of the OD curves (Fig. 4). The OD derivative of the WT or the mutant in point *i* was estimated to be (*OD*_*i*+*1*_*—OD*_*i*_*)/(time*_*i*+*1*_*—time*_*i*_*).* The p-values for the OD derivative and OD ratio was computed in as in the case of the OD p-value (described above) for single time points and ranges (entire log phase and entire stationary phase).

## Supplementary information

Supplementary Information.

Supplementary Information.
