# Supplementary material for: Algorithms for ribosome traffic engineering and their potential in improving host cells' titer and growth rate
Source: Sci Rep. 2020 Dec 3;10:21202. doi: 10.1038/s41598-020-78260-y (PMC7713304; doi:10.1038/s41598-020-78260-y)
Supplement: Supplementary file 2 — Supplementary Information. [file 41598_2020_78260_MOESM2_ESM.pdf]

Supplementary of the paper "Algorithms for Ribosome Traffic Engineering and their Potential in Improving Host Cells' Titer and Growth Rate"

Hadas Zur[1], Rachel Cohen-Kupiec[1], Sophie Vinokour[1], and Tamir Tuller[1,2]

[1]Department of Biomedical Engineering, the Engineering Faculty, Tel Aviv University.
[2]The Sagol School of Neuroscience, Tel Aviv University, Tel-Aviv 69978, Israel.
Contact: tamirtul@post.tau.ac.il (TT)

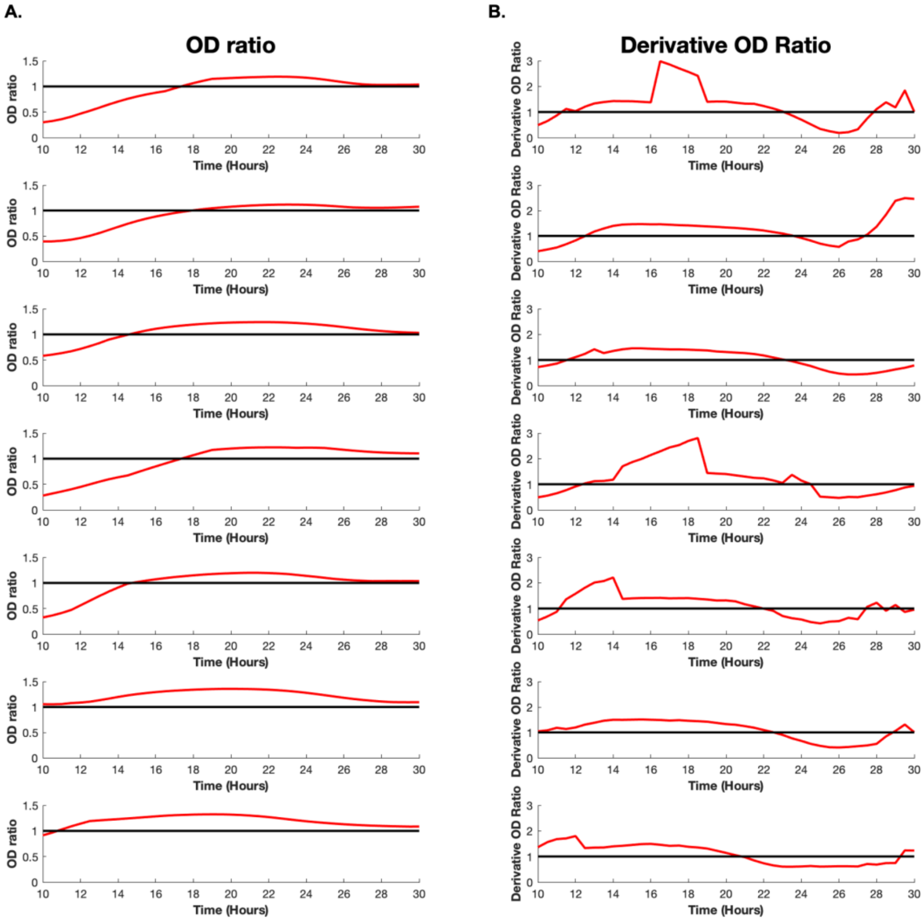

Figure S1: Plots of individual repeats of the double mutant and WT. A. The OD ratio between the mutant and the WT. B. The estimation of the ratio in the OD derivatives between mutant and the WT.

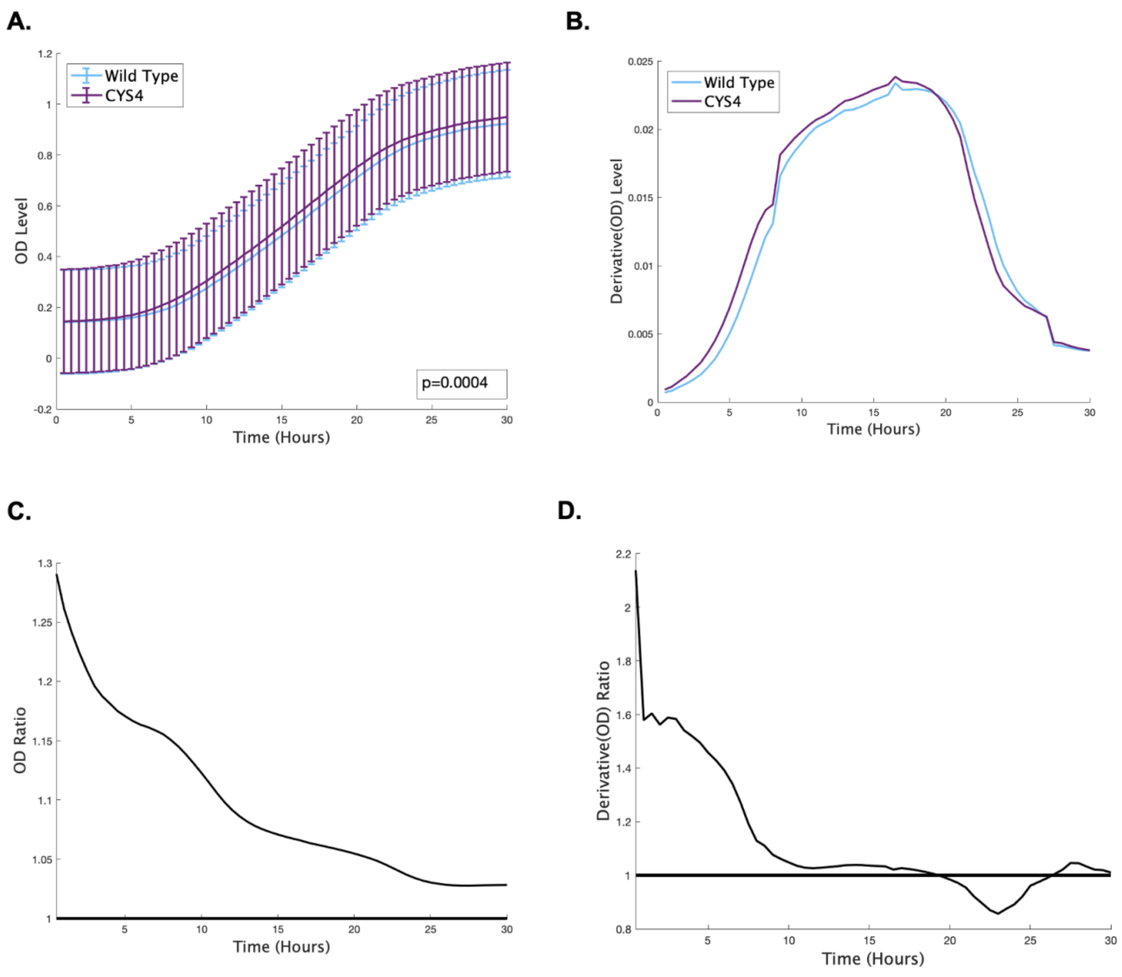

Figure S2. A. The titer, measured by OD, of the cys4 mutant is higher in all time points. The bars represent the STD in each point. B. Derivatives of the OD (i.e. the estimation of growth rate) of cys4 mutant and the WT. C. The OD ratio between the cys4 mutant and the WT. D. The estimation of the ratio in the OD derivatives between cys4 mutant and the WT.

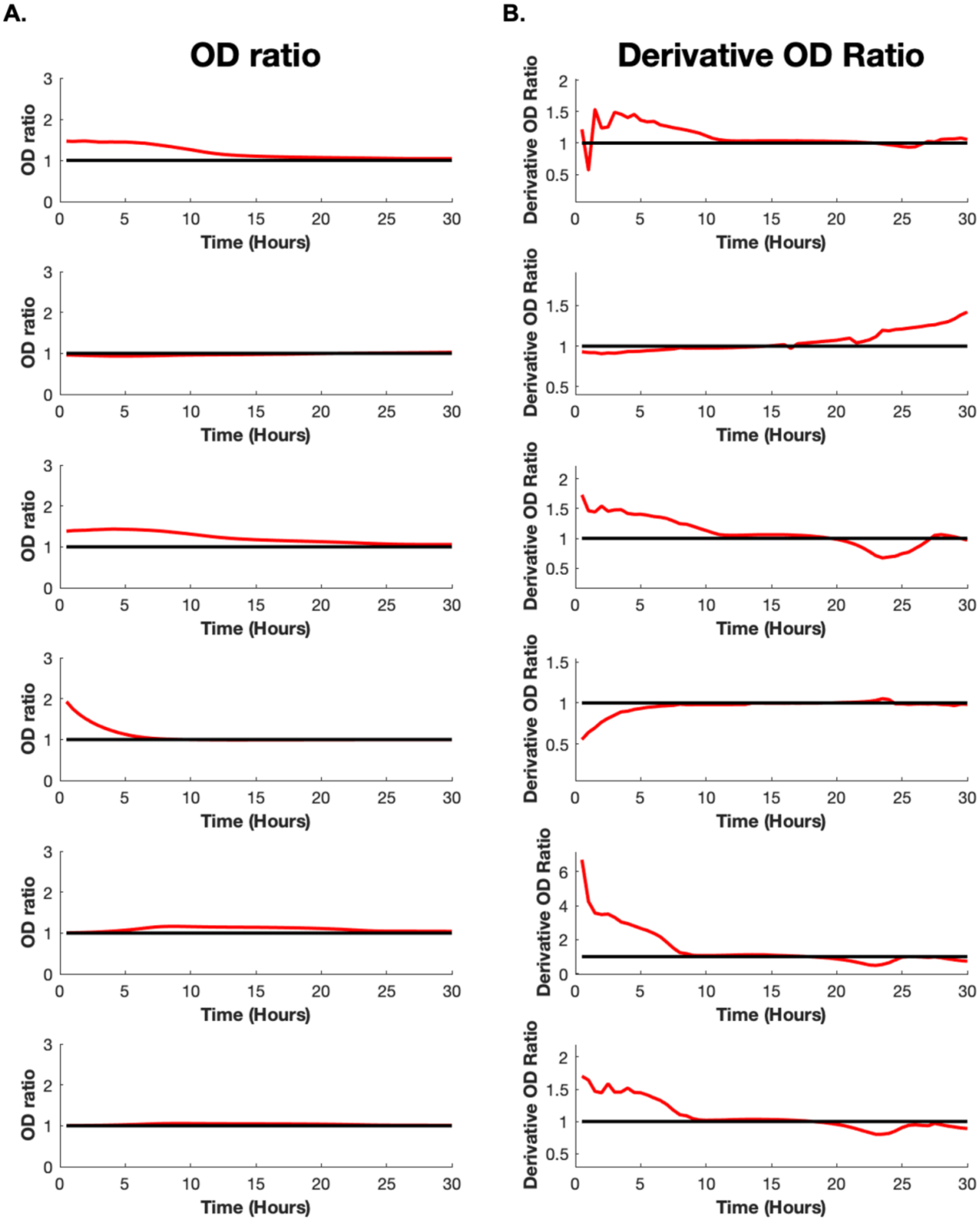

Figure S3: Plots of individual repeats of the cys4 mutant and WT. A. The OD ratio between the cys4 mutant and the WT. B. The estimation of the ratio in the OD derivatives between cys4 mutant and the WT.

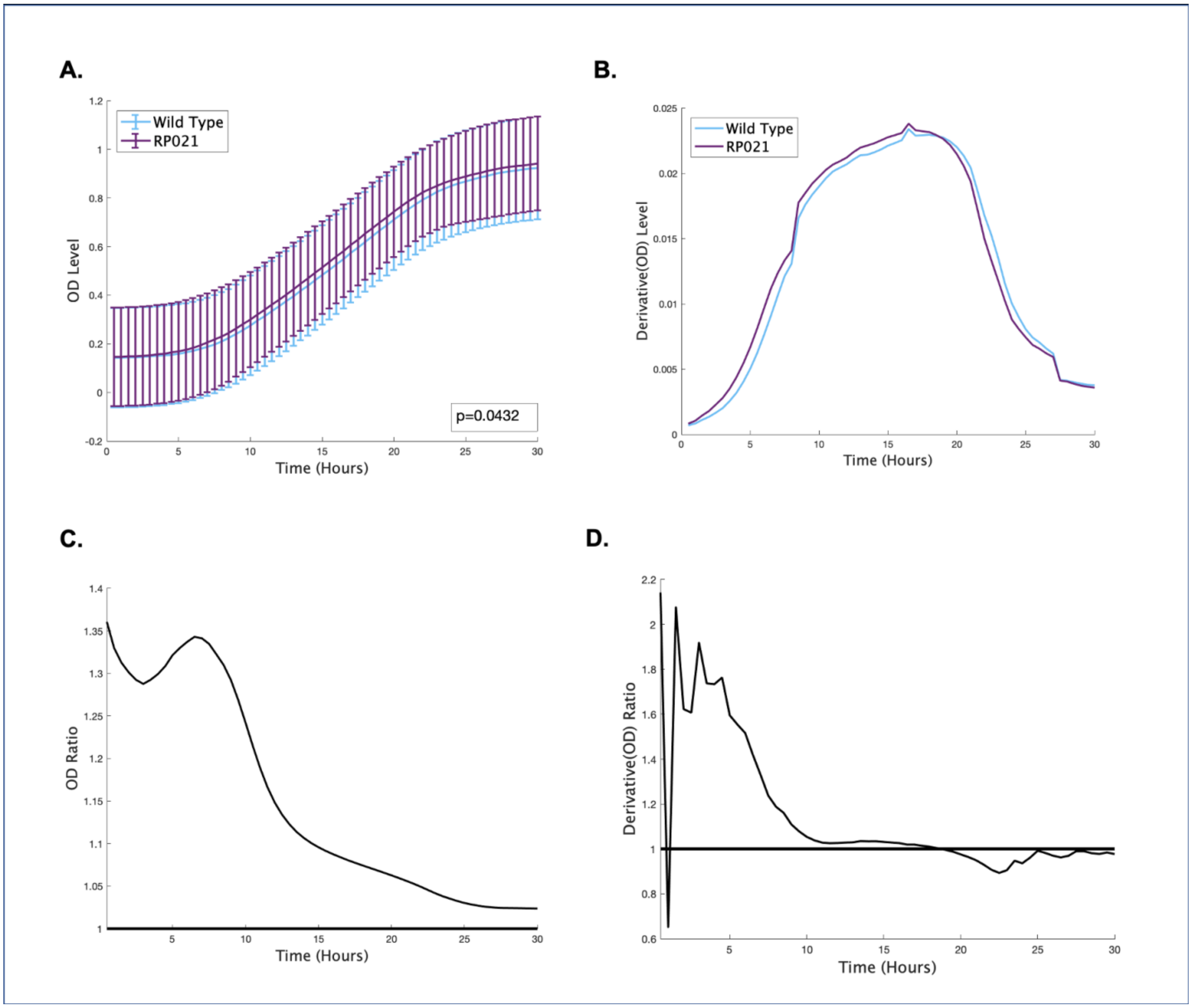

Figure S4. A. The titer, measured by OD, of the RP021 mutant is higher in all time points. The bars represent the STD in each point. B. Derivatives of the OD (i.e. the estimation of growth rate) of RP021 mutant and the WT. C. The OD ratio between the RP021 mutant and the WT. D. The estimation of the ratio in the OD derivatives between RP021 mutant and the WT.

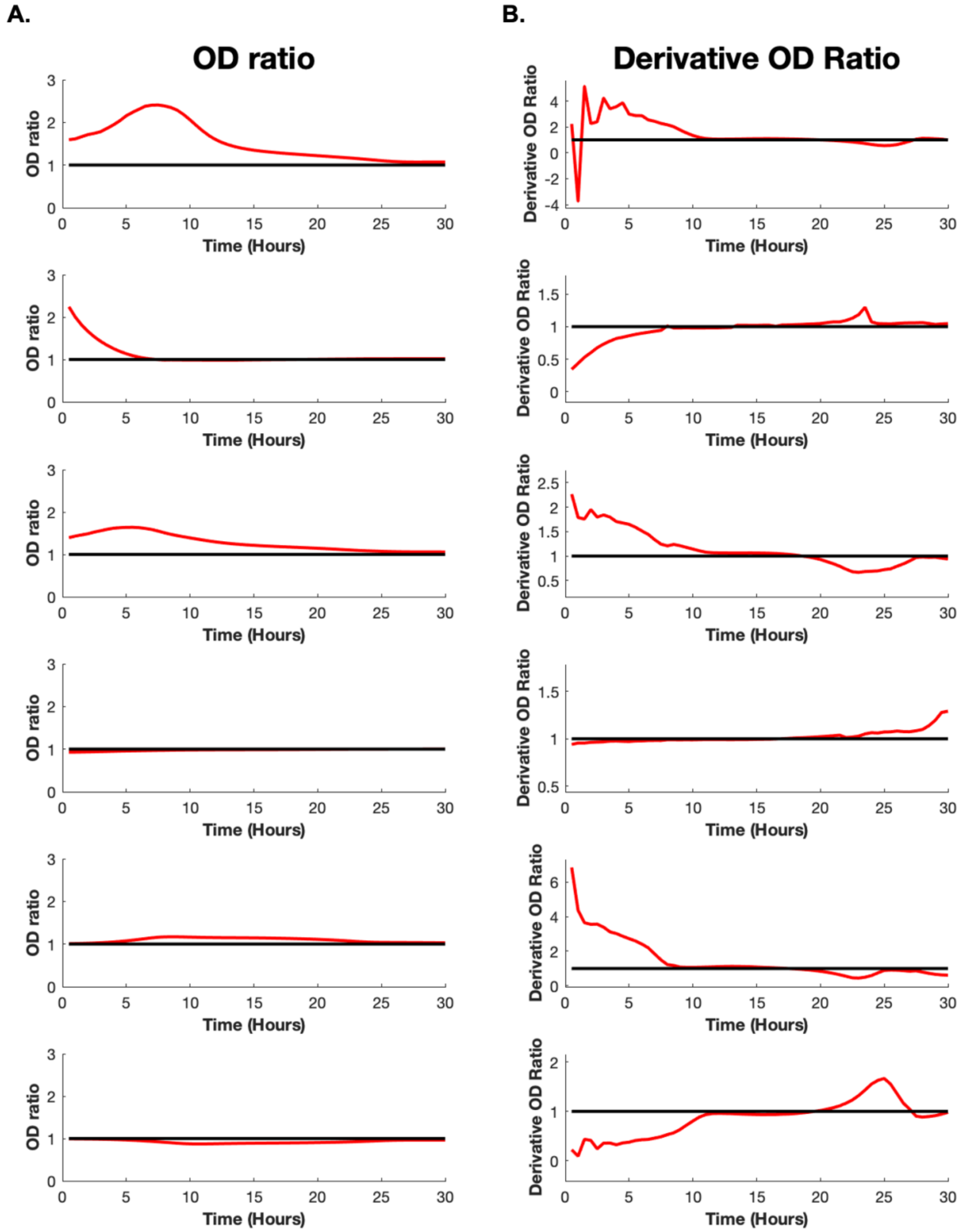

Figure S5: Plots of individual repeats of the RP021 mutant and WT. A. The OD ratio between the RP021 mutant and the WT. B. The estimation of the ratio in the OD derivatives between RP021 mutant and the WT.

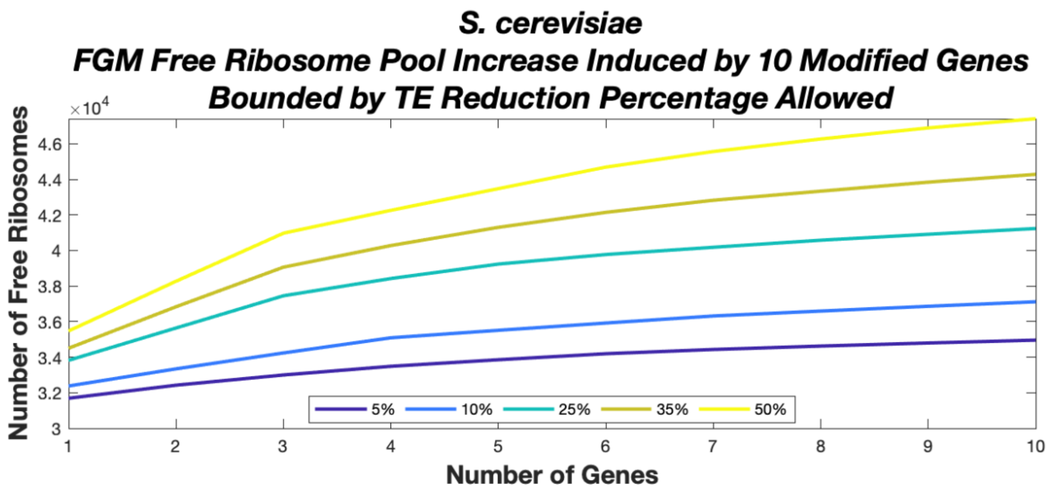

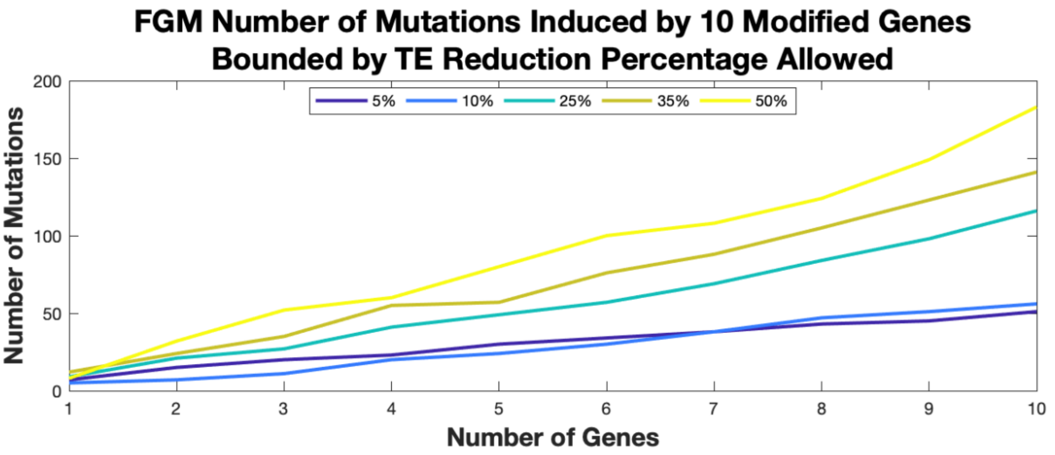

Figure S6 The performances of the algorithm in *S. cerevisiae* when allowing very large increase in TE of the mutated genes. A. The increase in the size of the free ribosomal pool. B. The number of mutations. As can be seen both the size of the pool and the number of mutations increases when allowing large increase in TE.

A.

Reduction in TE	FGM Free Ribosomes	BGM Free Ribosomes	GGM Free Ribosomes
**0.1%**	4111 (13.7%) [5.98]	3088 (10.29%) [4.65]	3183 (10.61%) [2.2]
**0.5%**	5118 (17.06%) [5.75]	4508 (15.03%) [4.61]	4333 (14.44%) [2.3]
**1%**	6012 (20.04%) [5.45]	5206 (17.35%) [4.97]	4890 (16.3%) [2.34]
**1.5%**	6662 (22.21%) [5.81]	5743 (19.14%) [5.13]	6063 (20.21%) [2.31]
**2%**	7261 (24.2%) [5.93]	6196 (20.65%) [5.44]	6616 (22.05%) [2.47]
**2.5%**	7783 (25.94%) [6.33]	6608 (22.03%) [5.69]	7154 (23.85%) [2.63]
**3%**	8380 (27.93%) [6.42]	7035 (23.45%) [5.83]	7823 (26.08%) [2.57]
**3.5%**	8946 (29.82%) [6.78]	7431 (24.77%) [5.88]	8135 (27.12%) [2.59]
**4%**	9529 (31.76%) [6.96]	7964 (26.55%) [5.9]	8575 (28.58%) [2.57]
**4.5%**	10024 (33.41%) [6.99]	8383 (27.94%) [6.31]	9131 (30.44%) [2.72]
**5%**	10517 (35.06%) [7.1]	8851 (29.5%) [6.41]	9490 (31.63%) [2.84]

B.

Reduction in TE	FGM Free Ribosomes	BGM Free Ribosomes	GGM Free Ribosomes
**0.1%**	890 (15.9%) [5.65]	935 (16.7%) [5.8]	972 (17.36%) [2.78]
**0.5%**	1554 (27.75%) [6.05]	1388 (24.79%) [5.91]	1525 (27.23%) [2.78]
**1%**	1815 (32.41%) [6.29]	1633 (29.16%) [6.29]	1794 (32.04%) [2.89]
**1.5%**	2091 (37.34%) [6.01]	1825 (32.59%) [6.59]	2008 (35.86%) [2.8]
**2%**	2261 (40.38%) [6.16]	1974 (35.25%) [6.81]	2195 (39.2%) [2.9]
**2.5%**	2471 (44.13%) [6.5]	2105 (37.59%) [7]	2362 (42.18%) [3.06]
**3%**	2631 (47%) [6.22]	2226 (39.75%) [7.01]	2536 (45.29%) [2.96]
**3.5%**	2775 (49.55%) [6.6]	2342 (41.82%) [7.84]	2685 (47.95%) [3.16]
**4%**	2916 (52.07%) [6.97]	2430 (43.39%) [7.64]	2794 (49.89%) [3.22]
**4.5%**	3061 (54.66%) [7.15]	2520 (45%) [7.93]	2927 (52.27%) [3.16]
**5%**	3199 (57.13%) [7.2]	2636 (47.07%) [8.04]	3067 (54.77%) [3.35]

Table S1. The table summarizes the additional number of free ribosomes made available by each of the three algorithms, for the top 100 genes respectively, per TE percentage reduction constraint in *S. cerevisiae (A.) and E. coli (B.)*, in parentheses the added percentage is specified, while in square brackets the mean number of mutations performed per gene.
